# Association between insulin resistance and sudomotor dysfunction in adults with type 1 diabetes

**DOI:** 10.1530/EC-25-0657

**Published:** 2025-12-02

**Authors:** Agnieszka Gandecka-Pempera, Aleksandra Uruska, Stanisław Piłaciński, Dorota Zozulińska-Ziółkiewicz, Aleksandra Araszkiewicz

**Affiliations:** Poznan University of Medical Sciences, Department of Internal Medicine and Diabetology, Poznan, Poland

**Keywords:** type 1 diabetes, insulin resistance, estimated glucose disposal rate, sudomotor function, small fiber neuropathy, diabetic complications

## Abstract

**Aims:**

Insulin resistance is increasingly recognized in type 1 diabetes as a risk factor for chronic complications and cardiovascular disease. Sudomotor dysfunction, reflecting small fiber neuropathy, can be assessed non-invasively by electrochemical skin conductance (ESC). This study evaluated the association between sudomotor function and insulin resistance in adults with type 1 diabetes.

**Methods:**

The study included 476 adults with type 1 diabetes (247 men), aged 42 (IQR: 33–53) years, with a disease duration of 24 (IQR: 19–32) years, and HbA1c level of 7.9 (IQR: 7.2–8.9)%. Sudomotor function was evaluated using the SUDOSCAN device. Insulin resistance was assessed using the estimated glucose disposal rate formula (eGDR), which is based on HbA1c, waist-to-hip ratio, and the presence of hypertension. The study group was subdivided into three groups based on tertiles of eGDR (<5.5, 5.5–9.5, >9.5 mg/kg/min).

**Results:**

Participants with lower eGDR (lower insulin sensitivity) had lower feet ESC 71 (IQR: 50–81) vs 79 (IQR: 63–85) vs 83 (IQR: 74–86) μS; *P* < 0.0001. We found a positive correlation between feet ESC and eGDR (Rs = 0.28, *P* < 0.001). In a multiple linear regression model, feet ESC was independently associated with eGDR (*β* = 0.16, *P* = 0.001).

**Conclusion:**

Insulin resistance is associated with impaired sudomotor function in adults with type 1 diabetes.

## Introduction

Type 1 diabetes (T1D) is a chronic autoimmune disease with an increasing incidence worldwide (1). Over the course of the disease, individuals are at risk of developing chronic complications, including both micro- and macrovascular pathologies, which may ultimately contribute to increased morbidity, disability, and mortality. Therefore, it is crucial to reduce modifiable risk factors associated with the development of these complications and to detect them at an early stage, when pathological changes may still be reversible.

Although insulin resistance is a well-recognized pathophysiological mechanism in type 2 diabetes (T2D), it is also present in many people with T1D (2, 3). Factors such as overweight, obesity, sedentary lifestyle, smoking, and high consumption of processed foods contribute to its development (4, 5, 6, 7). In addition, chronic high-dose exogenous insulin therapy may further promote insulin resistance (8, 9). Insulin resistance in T1D is an established risk factor not only for cardiovascular diseases, including coronary artery disease, heart failure, and atherosclerosis (10, 11, 12, 13), but also for the progression of neurovascular complications (14, 15, 16, 17, 18, 19, 20).

The gold standard for assessing insulin sensitivity is the hyperinsulinemic–euglycemic clamp (21), in which the glucose disposal rate (GDR) reflects peripheral tissue responsiveness to insulin (22). Due to its complexity, this method is not used in routine practice. Instead, the estimated glucose disposal rate (eGDR), calculated from clinical parameters including arterial hypertension (HT), waist-to-hip ratio (WHR), and glycated hemoglobin (HbA1c), offers a practical, validated alternative that correlates strongly with clamp results (23, 24, 25).

Impairment of sudomotor function – a manifestation of small fiber neuropathy – has been associated with microvascular complications in T1D (26) and is considered an early marker of diabetic neuropathy (27, 28). Sweat glands are innervated by thin, unmyelinated C-fibers, and their dysfunction can be assessed non-invasively by measuring electrochemical skin conductance (ESC). ESC provides a reproducible, operator-independent tool for diabetic foot ulcer risk assessment, improving early detection beyond monofilament testing (29). Moreover, sudomotor dysfunction has been correlated with subclinical carotid atherosclerosis in people with T2D (30). SUDOSCAN enhances cardiovascular risk assessment in T2D by accurately diagnosing microvascular complications, ensuring comprehensive patient evaluation (31). Recently, Anghel *et al.* highlighted its potential as a novel early screening biomarker for autonomic and cardiovascular complications (32).

### Objective

While evidence from studies in T2D indicates a relationship between sudomotor dysfunction and insulin resistance–related pathologies such as increased carotid intima–media thickness (IMT), this relationship has not yet been evaluated in people with T1D, despite the fact that many of them also exhibit increased insulin resistance. Therefore, the aim of the present study was to assess the association between insulin resistance and sudomotor function in adults with T1D.

## Subjects, materials and methods

### Subjects

A study was carried out in a T1D white adult cohort with at least 5 years of disease duration. All participants provided written informed consent before inclusion.

Exclusion criteria were: pregnancy, active foot ulceration or limb amputation in the past, acute infection, implanted electronic devices, mental and neurological disorders, and neuropathy due to causes other than diabetes (e.g., vitamin B12 deficiency, chronic alcohol use, or uncontrolled hypothyroidism). Our study conforms to the principles outlined in the Declaration of Helsinki and was approved by the local bioethics committee (no. 508/14).

The study group consisted of 476 consecutive individuals under the care of the department and was subdivided into three groups based on tertiles of eGDR (<5.5, 5.5–9.5, >9.5 mg/kg/min).

### Data collection

All study participants completed a questionnaire that collected information on age, sex, medical history, duration of diabetes, current treatment, and smoking status. Participants were considered smokers if they reported current smoking in the questionnaire. Each participant underwent a physical examination, including assessment of anthropometric parameters. Blood pressure was measured using the Korotkoff method in the seated position after a period of rest. Two measurements were taken 10 min apart, and hypertension (HT) was diagnosed if the average blood pressure exceeded 140/90 mmHg. Blood samples were collected in a fasting state, defined as no caloric intake for at least 8 h, using the S-Monovette blood collection system. HbA1c was assessed using a turbidimetric inhibition immunoassay (Cobas 6000; Roche Diagnostics), with a single measurement. Serum total cholesterol, HDL cholesterol, and triglyceride levels were measured with the Cobas 6000 biochemistry analyzer (Roche Diagnostics, Switzerland) using enzymatic colorimetric methods. LDL cholesterol level was calculated using the Friedewald formula. The serum concentration of C-reactive protein was measured using a highly sensitive method. Estimated GFR was calculated using the CKD-EPI formula. Diabetic kidney disease was diagnosed in participants with albumin-to-creatine ratio of at least 30 mg/g for longer than 3 months or eGFR of less than 60 mL/min/1.73 m^2^ (or both) in the absence of other primary causes of kidney injury (33). Diabetic retinopathy was diagnosed using fundoscopy after mydriasis. Peripheral neuropathy was detected using touch, vibration, and temperature sensation tests (34). The evaluation of cardiac autonomic neuropathy was performed using the ProsciCard III program (Medi-Syst GmbH, Germany).

### Skin autofluorescence assessment

Skin autofluorescence (AF) was measured with an AGE reader (type 214D00102; DiagnOptics, the Netherlands). Each test lasted 30 s and was performed at the ventral side of the forearm, about 5 cm distal to the elbow. This non-invasive device was used to assess the accumulation of advanced glycation end-products (AGEs) by exploiting fluorescent properties in tissues. AGE Reader has a source of ultraviolet radiation with a wavelength range of 300–420 nm and an optical spectrometer. AF is expressed as the ratio of the average intensity of light emitted in the wavelength range of 420–600 nm to the average light intensity in the wavelength range of 300–420 nm. For each participant, AF was measured three times in a series. The result is the arithmetic mean of these three measurements (35).

### Assessment of sudomotor function

The SUDOSCAN+ device (Impeto Medical, France) was used to assess sudomotor function. The device is composed of two pairs of stainless-steel electrodes, one pair dedicated to the hands and the other to the feet, connected to a computer for automated data processing. During the assessment, participants placed their palms and soles on the electrodes. The procedure is non-invasive, painless, and requires approximately 2 min. Throughout the test, a low-voltage direct current (<4 V) was applied, inducing an electrochemical reaction between sweat chloride ions and the electrodes. The resulting response is quantified as ESC, expressed in microsiemens (μS), calculated as the ratio of the generated current to the applied constant stimulus. Lower ESC values indicate impaired sudomotor function. According to the manufacturer’s reference data, normal values in women are 75 µS (IQR, 57–87 µS) for the hands and 83.5 µS (IQR, 71–90 µS) for the feet; in men, 76 µS (IQR, 56–88.5 µS) for the hands and 82.5 µS (IQR, 70–90.5 µS) for the feet (36, 37, 38). Participants were tested fasting, at room temperature, and at air humidity of 40–50%. Before testing, individuals were instructed to wash their hands and feet with soap and water.

### Insulin resistance measurement

Indirect measurements of insulin resistance, such as body mass index (BMI), waist circumference, waist–hip ratio (WHR), daily insulin requirement, and eGDR according to Williams *et al.* were assessed (23). We used the same medical scales to measure the height and weight of all participants. Height was measured to an accuracy of 0.5 cm and weight to 100 g. Waist and hip circumferences were assessed using a non-elastic tape to an accuracy of 1 mm. WHR was calculated as waist circumference (cm)/hip circumference (cm) and BMI as weight (kg)/height squared (m^2^). Insulin requirement was assessed as total daily insulin dose (U), expressed in units per kg body weight. Insulin sensitivity was evaluated with an equation for eGDR, modified for use with HbA1c instead of HbA 1 according to Thorn *et al.* (24) (eGDR = 24.4 − (12.97 × waist–hip ratio) − (3.39 × hypertension) − (0.60 × HbA1c)), where hypertension (yes = 1, no = 0) is defined if the participant was receiving antihypertensive medication or had systolic blood pressure >140 mmHg or diastolic blood pressure >90 mmHg, and the units of measurement are mg/kg/min. The cut-off value was set at 7.4 mg/kg/min, which was the median value for the whole study group according to hyperinsulinemic–euglycemic clamp studies (21); participants with eGDR <7.4 mg/kg/min were classified as insulin-resistant. The group was divided into three subgroups according to lower and upper tertiles of eGDR (below the first tertile <5.5 mg/kg/min; between the first and third tertiles 5.5–9 mg/kg/min; above the third tertile >9 mg/kg/min). The lower the eGDR, the lower the insulin sensitivity (23). It should be noted that the cutoff value of 7.4 mg/kg/min also represented the exact median eGDR of our study cohort, further supporting its use as a physiologically and statistically justified threshold.

### Statistical analysis

The statistical analysis was performed using the STATISTICA 13.0 program. The Kolmogorov–Smirnov test with Lilliefors correction was used to test for normality. Comparison of three groups divided by eGDR tertile was performed using ANOVA. The correlation between the conductivity of the skin and eGDR, clinical characteristics, metabolic control, and inflammatory markers was evaluated using the Spearman correlation coefficient. Multiple linear and logistic models were used to reveal the association between ESC and insulin resistance, assessed with eGDR. Variables were chosen on the basis of comparison of groups and in the stepwise model using data that were significantly different between groups with and without insulin resistance. Data included in the eGDR formula (HbA1c, waist–hip ratio, and presence of hypertension) were not analyzed in the regression model owing to redundancy. All data are expressed as medians and interquartile range or percentage of participants. Results with a probability value < 0.05 were considered statistically significant.

## Results

The study included 476 adults with T1D (247 men), age 42 (IQR: 33–53) years, with disease duration of 24 (IQR: 19–32) years and HbA1c level of 7.9 (IQR: 7.2–8.9) %. Among the study participants, 101 (21%) had diabetic kidney disease, 319 (67%) were diagnosed with diabetic retinopathy, 214 (45%) with peripheral neuropathy, and 95 (20%) with autonomic neuropathy. A total of 238 participants (50%) had lower insulin sensitivity, with eGDR <7.4 mg/kg/min. The clinical characteristics of the study group are shown in Table 1. Patients with lower eGDR (higher insulin resistance) had lower feet ESC (71 (IQR: 50–81) vs 79 (IQR: 63–85) vs 83 (IQR: 74–86) μS; *P* < 0.0001 and hands ESC (59 (IQR: 40–70) vs 61 (IQR: 50–73) vs 69 (IQR: 60–77) μS; *P* < 0.001)) (Table 2). It should be noted that, by definition, hypertension and higher WHR are integral components of the eGDR formula. Therefore, their uneven distribution across eGDR tertiles reflects a mathematical consequence of the score rather than an independent finding.

**Table 1:** Clinical characteristics of the study group (*n* = 476).

Characteristic	
Women/men (*n*) (%)	229 (48.1)/247 (51.9)
Age (years)	42 (33–53)
Duration of diabetes (years)	24 (19–32)
Smoker (*n*) (%)	133 (28)
Hypertension (*n*) (%)	237 (50)
Body weight (kg)	73 (63–84)
BMI (kg/m^2^)	25 (23–28)
WHR	0.87 (0.81–0.94)
Daily dose of insulin (u/kg b.w./24 h)	0.52 (0.41–0.64)
eGDR (mL/kg/min)	7.4 (5.4–9.4)
eGDR <7.4, (*n*) (%)	238 (50)
HbA1c (%)	7.9 (7.2–8.9)
HbA1c (mmol/mol)	63 (55–74)
TCh (mmol/L)	4.8 (4.1–5.7)
TG (mmol/L)	1.1 (0.8–1.4)
LDL (mmol/L)	3.0 (2.3–3.6)
HDL (mmol/L)	1.7 (1.4–2.0)
eGFR (mL/min/1.73 m^2^)	88.7 (74.8–101.8)
AF	2.5 (2.2–2.9)
hsCRP (mg/dL)	1.25 (0.6–2.6)
Hand ESC (μS)	63 (50–74)
Feet ESC (μS)	79 (64–84)
Diabetic kidney disease *n* (%)	101 (21%)
Retinopathy *n* (%)	319 (67%)
Peripheral neuropathy *n* (%)	214 (45%)
Autonomic neuropathy *n* (%)	95 (20%)

eGDR, estimated glucose disposal rate; BMI, body mass index; WHR, waist–hip ratio; TCh, total cholesterol; TG, triglycerides; LDL, low-density lipoprotein; HDL, high-density lipoprotein; eGFR, estimated glomerular filtration rate; AF, skin autofluorescence; ESC, electrochemical skin conductance. Values are median (interquartile range) or number and percentages.

**Table 2:** Comparison of characteristics according to estimated glucose disposal rate group (those below the lower tertile vs those between the first and third tertiles, and vs those above the third tertile), data presented as medians with interquartile ranges or as numbers and percentages (ANOVA test).

Characteristic	Group 1 eGDR <5.5 mg/kg/min	Group 2 eGDR 5.5–9.5 mg/kg/min	Group 3 eGDR >9.5 mg/kg/min	*P*
*n*	122	244	110	
Women/men (*n*) (%)	31 (25)/91 (75)	120 (49)/124 (51)	78 (71)/32 (29)	**<0.001**
Age (years)	48.5 (40–59)	41 (33–52.5)	34 (27–45)	**<0.001**
Duration of diabetes (years)	29 (22–34)	24 (18.5–30)	21.5 (16–30)	**<0.001**
Smoker (*n*) (%)	32 (26)	79 (32)	22 (20)	0.33
Hypertension (*n*) (%)	122 (100)	115 (47)	0 (0)	**<0.001**
Body weight (kg)	82 (71–93)	72 (62.8–83)	67.5 (61–74)	**<0.001**
BMI (kg/m^2^)	27.1 (24.7–30.3)	24.8 (22.6–27.9)	23.4 (22–25.2)	**<0.001**
WHR	0.95 (0.92–0.99)	0.86 (0.82–0.92)	0.79 (0.74–0.83)	**<0.001**
Daily dose of insulin (u/kg b.w./24 h)	0.54 (0.42–0.63)	0.53 (0.42–0.67)	0.50 (0.4–0.62)	0.42
HbA1c (%)	8.4 (7.6–9.0)	8.1 (7.2–9.2)	7.3 (6.7–7.8)	**<0.001**
TCh (mmol/L)	4.8 (4.2–5.9)	5.0 (4.0–5.7)	4.6 (4.1–5.5)	0.85
TG (mmol/L)	1.2 (1.0–1.7)	1.1 (0.9–1.5)	0.9 (0.7–1.1)	**<0.001**
LDL (mmol/L)	2.9 (2.4–3.8)	3.0 (2.4–3.6)	2.7 (2.2–3.3)	0.29
HDL (mmol/L)	1.6 (1.3–1.9)	1.6 (1.4–2.0)	1.9 (1.6–2.1)	**<0.001**
eGFR (mL/min/1.73 m^2^)	84 (72–95)	89 (75–105)	92 (84–104)	**0.02**
AF	2.5 (2.2–2.9)	2.3 (2.0–2.7)	2.1 (1.9–2.5)	**<0.001**
hsCRP (mg/dL)	1.8 (1.0–3.30	1.1 (0.6–2.4)	0.9 (0.5–2.1)	**0.001**
Hands ESC (μS)	59 (40–70)	61 (50–73)	69 (60–77)	**<0.001**
Feet ESC (μS)	71 (50–81)	79 (63–85)	83 (74–86)	**<0.001**
Diabetic kidney disease *n* (%)	43 (35%)	45 (18%)	13 (12%)	**<0.001**
Retinopathy *n* (%)	104 (85%)	165 (68%)	50 (46%)	**<0.001**
Peripheral neuropathy *n* (%)	76 (62%)	111 (45%)	27 (25%)	**<0.001**
Autonomic neuropathy *n* (%)	38 (31%)	46 (19%)	11 (10%)	**<0.001**

Bold indicates statistical significance, *P* < 0.05. eGDR, estimated glucose disposal rate; BMI, body mass index; WHR, waist–hip ratio; TCh, total cholesterol; TG, triglycerides; LDL, low-density lipoprotein; HDL, high-density lipoprotein; eGFR, estimated glomerular filtration rate; AF, skin autofluorescence; ESC, electrochemical skin conductance.

We found a positive correlation between feet and hands ESC and eGDR (Rs = 0.28, *P* < 0.001 and Rs = 0.21, *P* < 0.001, respectively) (Table 3). In a multiple linear regression model, which takes into account age, sex, and duration of diabetes, eGDR was independently associated with feet ESC (*β* = 0.16, *B* = 1.47 *P* < 0.001) (Table 4). To further evaluate the relative predictive strength of individual components of the eGDR, univariate linear regression analyses were performed for HbA1c, hypertension, WHR, and the composite eGDR (Table 5). Hypertension and HbA1c were both significantly and negatively associated with feet ESC (*β* = −0.22, *P* < 0.001 and *β* = −0.14, *P* = 0.003, respectively), whereas WHR did not reach statistical significance (*P* = 0.19). The composite eGDR, however, showed the strongest positive association with feet ESC (*β* = 0.23, *P* < 0.001), explaining the greatest proportion of variance (*R*^2^ = 0.06). These results indicate that the integrated eGDR index better reflects the relationship between insulin resistance and sudomotor dysfunction than any single clinical parameter alone. To further examine the relationship between insulin resistance and neuropathy severity, participants were stratified into three groups based on feet and hand ESC values: 1) normal feet and hand ESC (no neuropathy), 2) low feet ESC with normal hand ESC (length-dependent neuropathy), and 3) low feet and hand ESC (advanced neuropathy). Participants with normal feet ESC but low hand ESC were excluded from this analysis. Median eGDR values progressively declined across neuropathy stages: 8.2 (IQR: 5.9–9.9) mg/kg/min in group 1, 6.6 (IQR: 5.3–8.5) in group 2, and 6.1 (IQR: 4.9–8.0) mg/kg/min in group 3 (*P* < 0.001, Kruskal–Wallis test). This stepwise pattern indicates a dose–response relationship between insulin resistance and sudomotor dysfunction, suggesting that greater insulin resistance is associated with progressively worse neuropathy (Supplementary Table S1 (see section on Supplementary materials given at the end of the article)).

**Table 3:** Correlation between electrochemical skin conductance and clinical characteristics of the study group and estimated glucose disposal rate. Spearman’s coefficient.

Characteristic	Feet ESC		Hands ESC	
Variables	*R* value	*P*	*R* value	*P*
Age (years)	**−0.41**	**<0.001**	**−0.38**	**<0.001**
Duration of diabetes (years)	**−0.34**	**<0.001**	**−0.31**	**<0.001**
BMI (kg/m^2^)	0.03	0.46	0.09	0.04
WHR	−0.08	0.08	**−0.01**	**0.02**
Daily dose of insulin (u/kg b.w./24 h)	0.02	0.72	−0.0	0.98
eGDR (mL/kg/min)	**0.28**	**<0.001**	**0.21**	**<0.001**
HbA1c (%)	**−0.09**	**0.03**	**−0.09**	**0.04**
TCh (mmol/L)	**−0.15**	**0.02**	−0.12	0.06
TG (mmol/L)	**−0.18**	**<0.001**	**−0.13**	**0.004**
LDL (mmol/L)	−0.03	0.45	−0.0	0.98
HDL (mmol/L)	−0.03	0.47	−0.04	0.33
eGFR (mL/min/1.73 m^2^)	**0.39**	**<0.001**	**0.3**	**<0.001**
AF	**−0.37**	**<0.001**	**−0.32**	**<0.001**

Bold indicates statistical significance, *P* < 0.05.

**Table 4:** Regression coefficients of multiple linear regression with feet ESC as the dependent variable and sex, duration of diabetes, age, skin autofluorescence (AF), and estimated glucose disposal rate as independent variables, *R*^2^ = 0.2.

	*β*	*B*	*P*
Sex (male)	**0.14**	**2.9**	**0.002**
Duration of diabetes	0.02	0.05	0.69
Age	**−0.18**	**−0.29**	**0.003**
eGDR	**0.16**	**1.42**	**0.001**
AF	**−0.28**	**−7.09**	**<0.001**

Bold indicates statistical significance, *P* < 0.05.

**Table 5:** Regression coefficients from univariate linear models with feet ESC as the dependent variable and HbA1c, hypertension, WHR, and estimated glucose disposal rate (eGDR) as independent variables.

	*β*	*B*	*P*	*R* ^2^
HT	**−0.22**	**−4.7**	**<0.001**	**0.05**
WHR	−0.06	−14.3	0.19	0.004
HbA1c	**−0.14**	**−2.1**	**0.003**	**0.02**
eGDR	**0.23**	**2.15**	**<0.001**	**0.06**

Bold indicates statistical significance, *P* < 0.05.

The multiple logistic regression model showed a significant relationship between insulin resistance, assessed according to eGDR, and feet ESC (Table 6). A cut-off value of 70 µS for feet ESC was used in accordance with the manufacturer’s recommendation based on Vinik *et al.* study (39).

**Table 6:** Regression coefficients of multiple logistic regression with feet ESC (<70 µS) as dependent variable and sex, duration of diabetes, smoking, body weight, skin autofluorescence (AF), and estimated glucose disposal rate (eGDR) as independent variables.

	Odds ratio	95% CI	*P*
Duration of diabetes (years)	**1.03**	**1.0–1.05**	**0.03**
Body weight (kg)	1.0	0.98–1.02	0.89
eGDR (mg/kg/min)	**0.82**	**0.74–0.92**	**<0.001**
AF	**2.68**	**1.8–4.03**	**<0.001**
Sex (female)	**2.38**	**1.42–4.01**	**0.001**
Smoking (no)	1.1	0.67–1.8	0.71

Bold indicates statistical significance, *P* < 0.05.

## Discussion

To our knowledge, this is one of the first studies that exposed a significant association between insulin resistance described by eGDR and impaired sudomotor function measured by ESC in adult individuals with T1D. These results suggest that insulin resistance may contribute to dysfunction and degeneration of unmyelinated C fibers and the development of early-stage, often subclinical, neuropathy.

Insulin resistance is defined as a reduced biological response of target cells to insulin, despite normal or even elevated circulating levels of the hormone. Tissue resistance to insulin is influenced by both genetic and environmental factors. Lifestyle plays a pivotal role in the development of insulin resistance, particularly excessive caloric intake combined with insufficient physical activity – both of which contribute to visceral (abdominal) obesity – as well as cigarette smoking.

Insulin resistance is a characteristic feature of T2D and is strongly associated with obesity; however, it may also occur in individuals with T1D. In our study cohort, approximately 50% of participants were classified as insulin-resistant according to the predefined eGDR threshold of <7.4 mg/kg/min. This finding highlights the high prevalence of insulin resistance among individuals with T1D – a phenomenon traditionally associated with T2D – and is consistent with previous studies reporting its occurrence in 25–50% of T1D (40, 41, 42, 43). The observed 100% prevalence of hypertension in the lowest eGDR tertile arises from the structure of the formula itself, in which hypertension directly contributes to lower eGDR values. Therefore, this group should be interpreted as representing individuals with a severe metabolic syndrome–like phenotype rather than as a newly identified association between hypertension and insulin resistance. In addition, when the individual components of the eGDR formula (WHR, hypertension, and HbA1c) were examined separately, both hypertension and HbA1c showed significant but weaker associations with sudomotor function compared with the composite eGDR. The eGDR remained the strongest independent predictor of feet ESC, indicating that insulin resistance, as a bundled metabolic construct integrating multiple interrelated risk factors; more accurately reflects the underlying mechanisms of small-fiber dysfunction than any single component alone. Similarly, none of the broader anthropometric measures, including BMI, WHR, or total body weight, showed a significant independent association with feet ESC. This finding underscores that overall adiposity or body size alone cannot explain sudomotor dysfunction in T1D. Instead, the composite eGDR score, integrating glycemic control, hypertension, and fat distribution, more accurately reflects the metabolic milieu contributing to neural impairment.

Stratification according to feet and hand ESC categories revealed a stepwise decline in eGDR across neuropathy stages, consistent with a dose–response relationship between insulin resistance and sudomotor dysfunction. This suggests that increasing insulin resistance is associated with the transition from absence of neuropathy to length-dependent and, ultimately, advanced neuropathy in adults with T1D.

The association between eGDR and feet ESC was stronger than that observed for hand ESC, which is clinically expected in a length-dependent pattern of diabetic neuropathy. Distal nerves in the lower limbs are affected earlier and more severely, leading to greater sudomotor impairment in the feet. Therefore, feet ESC provides a more sensitive indicator of early small-fiber dysfunction and was used as the primary variable in our regression analyses. In T1D, insulin resistance arises from multiple mechanisms. Subcutaneous insulin administration bypasses the hepatic portal system, resulting in a weaker suppression of hepatic glucose production. Impaired glucose transporter function reduces skeletal muscle glucose uptake, while glucotoxicity and exogenous hyperinsulinemia further diminish peripheral insulin sensitivity in metabolically uncontrolled T1D (44, 45). Poor glycemic control exacerbates this state by enhancing hepatic glucose output and promoting lipolysis in adipocytes, increasing free fatty acid (FFA) flux to endothelial cells. Subsequent FFA oxidation drives reactive oxygen species (ROS) formation and protein kinase C (PKC) activation, contributing to neural ischemia, oxidative injury, and small fiber damage (46). In addition, our findings indicate that accumulation of advanced glycation end products (AGEs) in the skin – reflecting long-term metabolic control of diabetes – has an independent impact on sudomotor dysfunction, highlighting the role of chronic hyperglycemia–related protein modification in peripheral nerve impairment. Given that AGEs also impair insulin signaling through receptor-mediated oxidative stress and inflammatory pathways, their accumulation may represent a common mechanistic link between reduced sudomotor function and insulin resistance in T1D, thereby amplifying the risk of neuropathic progression (35, 47, 48).

Previously, the association between the Sudoscan cardiac risk score and metabolic syndrome has been described in the Chinese population, without distinguishing individuals with diabetes (49). In our study, sudomotor function was assessed using the Sudoscan device, which provides a non-invasive, rapid, and objective evaluation of small unmyelinated C-fiber function innervating sweat glands. Our results support its potential utility in identifying patients with subclinical nerve dysfunction who may benefit from intensified metabolic management. A potential improvement in sudomotor function has been reported in patients with T2D following bariatric intervention (50).

However, several limitations of the current study should be addressed. ESC values and diagnostic thresholds may vary depending on patient ethnicity. In our study, all participants were of Caucasian/European origin, resulting in an ethnically homogeneous group. While this reduces intra-cohort variability, it limits the generalizability of the results to populations of other ethnic backgrounds.

We used eGDR as a marker of insulin sensitivity; although it correlates strongly with hyperinsulinemic–euglycemic clamp results, it remains an indirect measure. Furthermore, while ESC is a sensitive marker of small-fiber neuropathy, confirmation with skin biopsies or more specific neurophysiological tests was not performed. On the other hand, applied methods are widely used in clinical and research approach, which makes these methods comparable and reliable. In addition, the use of non-invasive assessment methods is considered an important value of our study.

In conclusion, our findings indicate that insulin resistance is associated with impaired sudomotor function in adults with T1D, confirming the well-known role of metabolic dysregulation in the pathogenesis of diabetic neuropathy. The results highlight the importance of assessing insulin sensitivity even in patients with T1D as a potentially reversible risk factor for the development of diabetic complications.

## Supplementary materials



## Declaration of interest

The authors do not have any conflicts of interest to declare.

## Funding

This work did not receive any specific grant from any funding agency in the public, commercial, or not-for-profit sector.

## Ethics statement

The study was conducted in accordance with the Declaration of Helsinki. The study protocol was reviewed and approved by the Bioethical Committee of the Poznan University of Medical Sciences (approval no. 508/14). Written informed consent was obtained from all participants before study inclusion.

## References

[1] Liu J, Ren ZH, Qiang H, et al. Trends in the incidence of diabetes mellitus: results from the global burden of disease study 2017 and implications for diabetes mellitus prevention. BMC Public Health 2020 20 1415. (10.1186/s12889-020-09502-x) 32943028 PMC7500018

[2] Apostolopoulou M, Lambadiari V, Roden M, et al. Insulin resistance in type 1 diabetes: pathophysiological, clinical, and therapeutic relevance. Endocr Rev 2025 46 317–348. (10.1210/endrev/bnae032) 39998445 PMC12063105

[3] Kaul K, Apostolopoulou M & Roden M. Insulin resistance in type 1 diabetes mellitus. Metabolism 2015 64 1629–1639. (10.1016/j.metabol.2015.09.002) 26455399

[4] Polsky S & Ellis SL. Obesity, insulin resistance, and type 1 diabetes mellitus. Curr Opin Endocrinol Diabetes Obes 2015 22 277–282. (10.1097/med.0000000000000170) 26087341

[5] Mukharjee S, Bank S & Maiti S. Chronic tobacco exposure by smoking develops insulin resistance. Endocr Metab Immune Disord Drug Targets 2020 20 869–877. (10.2174/1871530320666200217123901) 32065107

[6] Jurkovičová J, Hirošová K, Vondrová D, et al. The prevalence of insulin resistance and the associated risk factors in a sample of 14–18-year-old Slovak adolescents. Int J Environ Res Public Health 2021 18 909. (10.3390/ijerph18030909) 33494341 PMC7908586

[7] Parker KM, Tucker LA, Bailey BW, et al. Relationship between sitting time and insulin resistance in 6931 U.S. adults: the mediating role of abdominal adiposity. J Diabetes Res 2023 2023 5015572. (10.1155/2023/5015572) 37265574 PMC10232095

[8] Gregory JM, Cherrington AD & Moore DJ. The peripheral peril: injected insulin induces insulin insensitivity in type 1 diabetes. Diabetes 2020 69 837–847. (10.2337/dbi19-0026) 32312900 PMC7171956

[9] Hamza SM, Sung MM, Gao F, et al. Chronic insulin infusion induces reversible glucose intolerance in lean rats yet ameliorates glucose intolerance in obese rats. Biochim Biophys Acta Gen Subj 2017 1861 313–322. (10.1016/j.bbagen.2016.11.029) 27871838

[10] Robins SJ, Rubins HB, Faas FH, et al. Insulin resistance and cardiovascular events with low HDL cholesterol: the veterans affairs HDL intervention trial (VA-HIT). Diabetes Care 2003 26 1513–1517. (10.2337/diacare.26.5.1513) 12716814

[11] Chen Q, Xiong S, Ye T, et al. Insulin resistance, coronary artery lesion complexity and adverse cardiovascular outcomes in patients with acute coronary syndrome. Cardiovasc Diabetol 2024 23 172. (10.1186/s12933-024-02276-1) 38755609 PMC11100181

[12] Czarnik K, Sablik Z, Borkowska A, et al. Insulin resistance may accelerate typical changes in heart function among type 1 diabetes patients, particularly in overweight patients: a preliminary study. Front Endocrinol 2024 15 1384514. (10.3389/fendo.2024.1384514) PMC11148266 38836221

[13] Fan Y, Yan Z, Li T, et al. Primordial drivers of diabetes heart disease: comprehensive insights into insulin resistance. Diabetes Metab J 2024 48 19–36. (10.4093/dmj.2023.0110) 38173376 PMC10850268

[14] Uruska A, Araszkiewicz A, Zozulinska-Ziolkiewicz D, et al. Insulin resistance is associated with microangiopathy in type 1 diabetic patients treated with intensive insulin therapy from the onset of disease. Exp Clin Endocrinol Diabetes 2010 118 478–484. (10.1055/s-0030-1249635) 20373280

[15] Sampath Kumar A, Arun Maiya G, Shastry BA, et al. Correlation between basal metabolic rate, visceral fat and insulin resistance among type 2 diabetes mellitus with peripheral neuropathy. Diabetes Metab Syndr 2019 13 344–348. (10.1016/j.dsx.2018.10.005) 30641723

[16] Xu YX, Pu SD, Zhang YT, et al. Insulin resistance is associated with the presence and severity of retinopathy in patients with type 2 diabetes. Clin Exp Ophthalmol 2024 52 63–77. (10.1111/ceo.14344) 38130181

[17] Liu Y, Peng Y, Jin J, et al. Insulin resistance is independently associated with cardiovascular autonomic neuropathy in type 2 diabetes. J Diabetes Investig 2021 12 1651–1662. (10.1111/jdi.13507) PMC8409868 33460512

[18] Linn W, Persson M, Rathsman B, et al. Estimated glucose disposal rate is associated with retinopathy and kidney disease in young people with type 1 diabetes: a nationwide observational study. Cardiovasc Diabetol 2023 22 61. (10.1186/s12933-023-01791-x) 36935526 PMC10024828

[19] Zooravar D, Radkhah H, Amiri BS, et al. Estimated glucose disposal rate and microvascular complications of diabetes mellitus type I: a systematic review and meta-analysis. Diab Vasc Dis Res 2025 22 14791641251324612. (10.1177/14791641251324612) 40114403 PMC11926832

[20] Parwani K & Mandal P. Chapter 5 - Advanced glycation end products and insulin resistance in diabetic nephropathy. Vitamins and Hormones 2024 125 117–148. (10.1016/bs.vh.2024.02.007) 38997162

[21] DeFronzo RA, Tobin JD & Andres R. Glucose clamp technique: a method for quantifying insulin secretion and resistance. Am J Physiol 1979 237 E214–E223. (10.1152/ajpendo.1979.237.3.e214) 382871

[22] Gastaldelli A. Measuring and estimating insulin resistance in clinical and research settings. Obesity 2022 30 1549–1563. (10.1002/oby.23503) 35894085 PMC9542105

[23] Williams KV, Erbey JR, Becker D, et al. Can clinical factors estimate insulin resistance in type 1 diabetes? Diabetes 2000 49 626–632. (10.2337/diabetes.49.4.626) 10871201

[24] Thorn LM, Forsblom C, Fagerudd J, et al. Metabolic syndrome in type 1 diabetes: association with diabetic nephropathy and glycemic control (the FinnDiane study). Diabetes Care 2005 28 2019–2024. (10.2337/diacare.28.8.2019) 16043748

[25] Januszewski AS, Niedzwiecki P, Sachithanandan N, et al. Independent euglycaemic hyperinsulinaemic clamp studies validate clinically applicable formulae to estimate insulin sensitivity in people with type 1 diabetes. Diabetes Metab Syndr Clin Res Rev 2023 17 102691. (10.1016/j.dsx.2022.102691) 36508938

[26] Gandecka A, Araszkiewicz A, Piłaciński S, et al. Evaluation of sudomotor function in adult patients with long-lasting type 1 diabetes. Pol Arch Intern Med 2017 127 16–24. (10.20452/pamw.3884) 28075427

[27] Casellini CM, Parson HK, Richardson MS, et al. Sudoscan, a noninvasive tool for detecting diabetic small fiber neuropathy and autonomic dysfunction. Diabetes Technol Ther 2013 15 948–953. (10.1089/dia.2013.0129) 23889506 PMC3817891

[28] Gin H, Baudoin R, Raffaitin CH, et al. Non-invasive and quantitative assessment of sudomotor function for peripheral diabetic neuropathy evaluation. Diabetes Metab 2011 37 527–532. (10.1016/j.diabet.2011.05.003) 21715211

[29] Gautier JF, Riveline JP, Potier L, et al. Electrochemical skin conductance: a tool for risk stratification and early anticipation of diabetic foot ulcers. Front Endocrinol 2025 16 1437858. (10.3389/fendo.2025.1437858) PMC11955488 40166674

[30] Wang M, Lu J, Zhu W, et al. Association of sudomotor dysfunction with risk of subclinical carotid atherosclerosis in patients with type 2 diabetes. Diabetes Metab Res Rev 2025 41 e70030. (10.1002/dmrr.70030) 39748556

[31] Figueroa-Perez CA, Romero-Ibarguengoitia ME, Garza-Silva A, et al. Sudoscan® reclassifies cardiovascular risk in patients with type 2 diabetes mellitus according to the ESC 2023. J Diabetes Metab Disord 2025 24 50. (10.1007/s40200-024-01548-7) 39845906 PMC11748664

[32] Anghel L, Cobuz C, Benchea LC, et al. Sudomotor dysfunction as an early marker of autonomic and cardiovascular risk in diabetes: insights from a cross-sectional study using SUDOSCAN. Biosensors 2025 15 372. (10.3390/bios15060372) 40558453 PMC12191207

[33] Kidney Disease: Improving Global Outcomes (KDIGO) CKD Work Group. KDIGO 2024 clinical practice guideline for the evaluation and management of chronic kidney disease. Kidney Int 2024 105 S117–S314. (10.1016/j.kint.2023.10.018) 38490803

[34] American Diabetes Association Professional Practice Committee. 12. Retinopathy, neuropathy, and foot care: standards of care in diabetes – 2024. Diabetes Care 2023 47 (Supplement_1) S231–S243. (10.2337/dc24-s012) PMC10725803 38078577

[35] Uruska A, Gandecka A, Araszkiewicz A, et al. Accumulation of advanced glycation end products in the skin is accelerated in relation to insulin resistance in people with type 1 diabetes mellitus. Diabet Med 2019 36 620–625. (10.1111/dme.13921) 30706538

[36] Yajnik CS, Kantikar VV, Pande AJ, et al. Quick and simple evaluation of sudomotor function for screening of diabetic neuropathy. ISRN Endocrinol 2012 2012 103714. (10.5402/2012/103714) 22830040 PMC3399356

[37] Yajnik CS, Kantikar V, Pande A, et al. Screening of cardiovascular autonomic neuropathy in patients with diabetes using non-invasive quick and simple assessment of sudomotor function. Diabetes Metab 2013 39 126–131. (10.1016/j.diabet.2012.09.004) 23159130

[38] Gordon Smith A, Lessard M, Reyna S, et al. The diagnostic utility of sudoscan for distal symmetric peripheral neuropathy. J Diabetes Complications 2014 28 511–516. (10.1016/j.jdiacomp.2014.02.013) 24661818 PMC4219320

[39] Vinik AI, Nevoret ML & Casellini C. The new age of sudomotor function testing: a sensitive and specific biomarker for diagnosis, estimation of severity, monitoring progression, and regression in response to intervention. Front Endocrinol 2015 6 94. (10.3389/fendo.2015.00094) PMC4463960 26124748

[40] Cleland SJ, Fisher BM, Colhoun HM, et al. Insulin resistance in type 1 diabetes: what is ‘double diabetes’ and what are the risks? Diabetologia 2013 56 1462–1470. (10.1007/s00125-013-2904-2) 23613085 PMC3671104

[41] Teixeira MM, Diniz MDFHS, Reis JS, et al. Insulin resistance and associated factors in patients with type 1 diabetes. Diabetol Metab Syndr 2014 6 131. (10.1186/1758-5996-6-131) 25937839 PMC4416245

[42] Šimonienė D, Platūkiene A, Prakapienė E, et al. Insulin resistance in type 1 diabetes mellitus and its association with patient’s micro- and macrovascular complications, sex hormones, and other clinical data. Diabetes Ther 2020 11 161–174. (10.1007/s13300-019-00729-5) 31792784 PMC6965600

[43] Kilpatrick ES, Rigby AS & Atkin SL. Insulin resistance, the metabolic syndrome, and complication risk in type 1 diabetes: ‘double diabetes’ in the diabetes control and complications trial. Diabetes Care 2007 30 707–712. (10.2337/dc06-1982) 17327345

[44] Sammut MJ, Dotzert MS & Melling CWJ. Mechanisms of insulin resistance in type 1 diabetes mellitus: a case of glucolipotoxicity in skeletal muscle. J Cell Physiol 2024 239 e31419. (10.1002/jcp.31419) 39192756 PMC11649966

[45] Okamoto MM, Anhê GF, Sabino-Silva R, et al. Intensive insulin treatment induces insulin resistance in diabetic rats by impairing glucose metabolism-related mechanisms in muscle and liver. J Endocrinol 2011 211 55–64. (10.1530/JOE-11-0105) 21746792

[46] Lipke K, Kubis-Kubiak A & Piwowar A. Molecular mechanism of lipotoxicity as an interesting aspect in the development of pathological states – current view of knowledge. Cells 2022 11 844. (10.3390/cells11050844) 35269467 PMC8909283

[47] Araszkiewicz A, Gandecka A, Nowicki M, et al. Association between small fiber neuropathy and higher skin accumulation of advanced glycation end products in patients with type 1 diabetes. Pol Arch Med Wewn 2016 126 847–853. (10.20452/pamw.3649) 27906877

[48] Araszkiewicz A, Naskret D, Niedzwiecki P, et al. Increased accumulation of skin advanced glycation end products is associated with microvascular complications in type 1 diabetes. Diabetes Technol Ther 2011 13 837–842. (10.1089/dia.2011.0043) 21568748

[49] Zhu L, Zhao X, Zeng P, et al. Study on autonomic dysfunction and metabolic syndrome in Chinese patients. J Diabetes Investig 2016 7 901–907. (10.1111/jdi.12524) PMC5089954 27181217

[50] Casellini CM, Parson HK, Hodges K, et al. Bariatric surgery restores cardiac and sudomotor autonomic C-Fiber dysfunction towards normal in obese subjects with type 2 diabetes. PLoS One 2016 11 e0154211. (10.1371/journal.pone.0154211) 27137224 PMC4854471

