# Supplementary material for: Association between insulin resistance and sudomotor dysfunction in adults with type 1 diabetes
Source: Endocr Connect. 2025 Dec 2;14(12):e250657. doi: 10.1530/EC-25-0657 (PMC12678851; doi:10.1530/EC-25-0657)
Supplement: Supplementary file 1 [file supplementary_materials.pdf]

Supplementary Table S1. eGDR values according to neuropathy stage based on Feet and Hand ESC categories (Kruskal–Wallis test). Data are expressed as medians (interquartile range).

Variable	Group 1 (No Neuropathy) Normal Feet ESC and Normal Hand ESC	Group 2 (Length-Dependent Neuropathy) Poor Feet ESC/Normal Hands ESC	Group 3 (Advanced Neuropathy) Poor Feet ESC/Poor Hand ESC	p
n	243	33	121	
eGDR [ml/kg/min]	8.2 (5.9-9.9)	6.6 (5.3-8.5)	6.1 (4.9-8.0)	<0.001
